# Inferential procedures based on the weighted Pearson correlation coefficient test statistic

**DOI:** 10.1080/02664763.2022.2137477

**Published:** 2022-10-25

**Authors:** Han Yu, Alan D. Hutson

**Affiliations:** Department of Biostatistics and Bioinformatics, Roswell Park Cancer Institute, Buffalo, NY, USA

**Keywords:** Permutation test, type i error control, linear association, computational methods, small sample

## Abstract

In this note, we evaluated the type I error control of the commonly used *t*-test found in most statistical software packages for testing the hypothesis on $H_{0}:\rho =0$ vs. $H_{1}:\rho >0$ based on the sample weighted Pearson correlation coefficient. We found the type I error rate is severely inflated in general cases, even under bivariate normality. To address this issue, we derived the large sample variance of the weighted Pearson correlation. Based on this result, we proposed an asymptotic test and a set of studentized permutation tests. A comprehensive set of simulation studies with a range of sample sizes and a variety of underlying distributions were conducted. The studentized permutation test based on Fisher's *Z* statistic was shown to robustly control the type I error even in the small sample and non-normality settings. The method was demonstrated with an example data of country-level preterm birth rates.

## 1. Introduction

To date, no valid procedure has been proposed for the inference of Pearson correlation coefficient based on a weighted Pearson correlation statistic. The commonly used statistical software such as SAS (SAS Institute Inc., Cary, NC, USA) does not provide valid inference results for weighted Pearson correlations. Built upon the methods by DiCiccio [5] and [12], we derived the large sample variance of the weighted Pearson correlation and proposed a set of studentized permutation tests. We will show the proposed tests can achieve robust the type I error control even when the sample size is small and the data does not follow normal distributions.

Throughout this note, let $\left(X_{1},Y_{1}),(X_{2},Y_{2}),\ldots ,(X_{n},Y_{n}\right)$ be *n* paired observations from a non-degenerate joint distribution $F_{\mathrm{XY}}(x,y)$. The weighted sample Pearson correlation estimator for *ρ* and fixed weights is given as

(1) $${\hat{\rho }}_{w}=\frac{\sum_{i=1}^{n}w_{i}(X_{i}-{\bar{X}}_{w})(Y_{i}-{\bar{Y}}_{w})}{\sqrt{\sum_{i=1}^{n}w_{i}(X_{i}-{\bar{X}}_{w}{)}^{2}}\sqrt{\sum_{i=1}^{n}w_{i}(Y_{i}-{\bar{Y}}_{w}{)}^{2}}},$$

where ${\bar{X}}_{w}=\sum_{i=1}^{n}w_{i}X_{i}$, $\bar{Y}=\sum_{i=1}^{n}w_{i}Y_{i}$, $\sum_{i=1}^{n}w_{i}=1$ and $0<w_{i}<1,i=1,2,\ldots ,n$. When all $w_{i}=1/n$ the estimator at (1) reduces to the classic sample Pearson correlation estimator, which we will denote as $\hat{\rho }$. In practice, the weights often reflect the relative importance of each observation. For example, it can be the population size if we are interested in the correlation of population-level measurements. The weights may also reflect the possibility of an observation of being an outlier or influential data point if a robust estimation is of utmost importance. Although the sample weighted and unweighted correlations will have different values, we emphasize that, in our setting, both are consistent estimates of the same population correlation coefficient.

Historically for the case when all $w_{i}=1/n$ the majority of statistical software packages, both commerical and freely available, utilize the oftentimes unrealistic underlying assumption of bivariate normality for testing $H_{0}:\rho =0$. Investigation of the test's robustness to the distribution assumption can be traced back to E.S. Pearson [17], who has concluded that the distribution of $\hat{\rho }$ is robust to ‘mutilation’ and ‘distortion’ to a remarkable degree, and a similar erroneous conclusion was drawn by later investigators as well [10]. In fact, extensive simulation-based studies, especially in the area of psychology, have noted that the tests tend to fail in type I error control when the bivariate normality assumption does not hold (e.g. [1–4,6]).

More recently, one can find examples of what we term the ‘naive permutation’ test for testing $H_{0}:\rho =0$, which consists of permuting either the column of *X*'s or *Y*'s and comparing $\hat{\rho }$ to the tail of the permutation distribution of the permuted $\hat{\rho }$'s to obtain a one-sided *p* -value. However, what is often overlooked in this testing scenario is that the assumption of *exchangeability* is often not satisfied, i.e. the permutation test of $H_{0}:\rho =0$ is only exact when the hypothesis is equivalent to $H_{0}:F_{\mathrm{XY}}=F_{X}F_{Y}$, which only holds under limited distribution assumptions, such as bivariate normality. Even though the permutation tests of correlation coefficients have been studied for decades [11], mistakes regarding its applicability under non-normality have been constantly made, even in recent works [22].

Recently, DiCiccio and Romano [5] provided a thorough discussion of this subject and proposed a studentized permutation test that asymptotically controls the type I error for testing $H_{0}:\rho =0$. The test robustly controls type I error even when the exchangeability assumption does not hold, which is achieved by studentizing the test statistic $\hat{\rho }$ by its large sample variance under $H_{0}$. The standardization ensures the test statistic's permutation distribution and sampling distributions both converge to the corresponding quantiles of the standard normal distribution. Such form of the standardization was first utilized by Tschuprow *et al.* (1925) for testing $H_{0}:\rho =0$. On the other hand, most of the commonly used tests for testing $H_{0}:\rho =0$, as implemented in many statistical software packages, strongly rely on the bivariate normality assumption, which is rarely satisfied in real-life data analysis problems [9]. Recently, Yu and Hutson [23] developed the R package *perk* available at github for calculating the appropriate Pearson correlation permutation test based on the work of [5]. In addition, this package accommodates the permutation test for the Spearman correlation and the concordance correlation coefficient based on the same principles. More recently, Hutson [12] derived a bootstrap approach to test the more general hypothesis $H_{0}:\rho =\rho_{0}$, which for the specific case of $H_{0}:\rho =0$ generally outperforms the permutation of [5] in terms of the desired Type I error control.

Using the above as a stepping-off point we now focus on the weighted estimator at (1). There are a variety of reasons provided for utilizing the weighted Pearson correlation from incorporating weights as a function of the time data was collected to downweighting extreme observations. Weights may be fixed or random. The random versus fixed weight scenarios need to be treated as distinct cases. Currently, inference about $H_{0}:\rho =0$ when using the test statistic ${\hat{\rho }}_{w}$ at (1) is sketchy at best and oftentimes completely wrong in routines found in commercial and freely available software. For example, PROC CORR, SAS 9.4 ( SAS Institute Inc., Cary, NC, USA), provides a *p*-value for the test $H_{0}:\rho =0$ assuming bivariate normality and utilizing the *t*-distribution as per [8]. However, when the WEIGHT statement is used SAS provides an incorrect *p*-value assuming ${\hat{\rho }}_{w}$ follows the same underlying distribution as $\hat{\rho }$, which is not the case due to a different variance between the two estimators, which in turn leads to a biased test with dramatically increased Type I error rates. Furthermore, SAS also provides a *p*-value via Fisher's *z*-transformation for the more general hypothesis $H_{0}:\rho =\rho_{0}$ for which they state in their online support that ‘Furthermore, even the distribution of *z* is not strictly normal, it tends to normality rapidly as the sample size increases …’, which [5] clearly illustrate is not true via a detailed simulation study contained in their supplemental material for the specific test $H_{0}:\rho =0$. In fact, [5] show that the test based on Fisher's *z*-transform behaves similarly and poorly in a variety of scenarios to that based on the *t*-distribution version of the test. These errors are compounded when a weighting scheme is considered. Similar errors about testing $H_{0}:\rho =0$ when using the test statistic ${\hat{\rho }}_{w}$ at (1) are found across a variety of freely available R packages.

Interestingly, for the case of equal weights very little attention or study has been given to the large sample approximation for the sampling distribution of $\hat{\rho }$, which does not rely on bivariate normality and is essentially ‘distribution free’. The asymptotic distribution can be derived using the delta method and can be found in [19], which was utilized in the recent work by Hutson [12]. Here we give a brief description of the derivation. Firstly, we note that the sample estimator can be expressed as $\hat{\rho }=g(V)$, where

(2) $$V=\left(\bar{X},\bar{Y},\frac{1}{n}\sum_{i=1}^{n}X_{i}^{2},\frac{1}{n}\sum_{i=1}^{n}Y_{i}^{2},\frac{1}{n}\sum_{i=1}^{n}X_{i}Y_{i}\right),$$

such that we have

(3) $$\hat{\rho }∼\mathrm{AN}(\rho ,n^{-1}\mathrm{d\Sigma}d^{\prime }).$$

For expression (3) Σ is the 5×5 variance-covariance matrix for the vector of the components of the summands of V given by $\left(X_{1},Y_{1},X_{1}^{2},Y_{1}^{2},X_{1}Y_{1}\right)$. The elements of d are given as

(4) $$\begin{array}{r} d_{1}=\frac{\rho \mu_{X}}{\sigma_{X}^{2}}-\frac{\mu_{Y}}{\sigma_{X}\sigma_{Y}},d_{2}=\frac{\rho \mu_{Y}}{\sigma_{Y}^{2}}-\frac{\mu_{X}}{\sigma_{X}\sigma_{Y}},\\ d_{3}=-\frac{\rho }{2\sigma_{X}^{2}},d_{4}=-\frac{\rho }{2\sigma_{Y}^{2}},d_{5}=\frac{1}{\sigma_{X}\sigma_{Y}}. \end{array}$$

**Comment.** As is well-known for the specific case that $F_{\mathrm{XY}}$ is bivariate normal (3) simplifies to $\hat{\rho }∼\mathrm{AN}(\rho ,n^{-1}(1-\rho^{2}{)}^{2})$.

An estimator for the variance of $\hat{\rho }$ may be given by substituting the respective moment estimators into (4). Hutson [12] illustrated that for samples of size *n*>50 this approximate worked well in terms of type I error control for testing the general hypothesis $H_{0}:\rho =\rho_{0}$.

In this note, we combine the elements of large sample theory and the methodology of [5]. In Section 2 we provide the large sample approximation for the distribution of ${\hat{\rho }}_{w}$ for both fixed and random weights. In Section 3 we extend the approaches of DiCiccio and Romano [5] and Hutson [12] for tests of $H_{0}:\rho =0$ using the weighted Pearson correlation test statistic. In Section 6 a real-world example on the correlation of country-level preterm with stillbirth and neonatal death rates was used to demonstrate the proposed tests. Overall, we will show that the proposed test is highly robust in type I error control and can lead to different conclusions in real-world data analysis. The routine usage of *t*-test for the inference of weighted Pearson correlation coefficient needs to be carefully re-examined.

## 2. Asymptotic distribution for the sample weighted Pearson correlation

In this section, we provide results for the large sample theory for ${\hat{\rho }}_{w}$ at (1) for both the fixed and random weight settings.

### 2.1. Fixed weights

In terms of preliminaries, we start with the work of Fisher [7] re-written in order to accommodate our notation. For the sequence of real numbers $\left\{w_{n}:n=1,2,3,\ldots \right\}$ define $W_{n}^{2}=\sum_{i=1}^{n}w_{i}^{2}$ and define the weighted sum of i.i.d. random variables $S_{n}=\sum_{i=1}^{n}w_{i}X_{i}$, where E(X)=0 and $E(X^{2})=1$. Let $T_{n}=\sum_{i=1}^{n}w_{i}^{2}V_{i}$, where $\left\{V_{n}:n=1,2,3,\ldots \right\}$ is a sequence of i.i.d. random variables such that $V_{i}>0$ and E(V)=1. Then the strong law holds if

(5) $$T_{n}/W_{n}^{2}\to 1\;a.s.\;\mathrm{as}\;n\;\to \infty .$$

Then we have

Lemma 2.1 **Fisher [7]**

Sufficient conditions for the strong law  (5), to hold are that

(6) $$\begin{array}{rl} W_{n}^{2} & \to \infty \;\;\text{and}\\ \;\;\;\;\;\text{\{Condition A:\}}\;w_{n}^{2}/W_{n}^{2} & =O\left(\frac{1}{n}\right). \end{array}$$

This yields

Theorem 2.1 **[7]**

As $W_{n}^{2}↑\infty$ and condition  (6) holds, then

(7) $$S_{n}/W_{n}\to Z\;\mathrm{weakly}\;\mathrm{as}\;n\to \infty ,$$

where Z is a standard normal random variable. Condition A at  (6) is described in detail in [7]. It refers to the class of sequences for $w_{n}$ such that the above Theorem holds. Our restriction that $\sum_{i=1}^{n}w_{i}=1$ and $0<w_{i}<1,i=1,2,\ldots ,n$ satisify ‘Condition A’ at  (6). Given the preliminary theory worked out by [7] we now have the following:

Theorem 1 The asymptotic distribution for ${\hat{\rho }}_{w}$ defined at  (1) for large *n* is given as

(8) $${\hat{\rho }}_{w}∼\mathrm{AN}(\rho ,\sum_{i=1}^{n}w_{i}^{2}d\Sigma d^{\prime }),$$

where the elements of vector d are given at (4) and Σ is the 5×5 variance-covariance matrix for the vector of the components of the summands of V defined at  (2) given by $\left(X_{1},Y_{1},X_{1}^{2},Y_{1}^{2},X_{1}Y_{1}\right)$.

Proof. Following a somewhat similar approach found in [19] for $\hat{\rho }$ first note that ${\hat{\rho }}_{w}$ may be written in the form

(9) $${\hat{\rho }}_{w}=\frac{\sum_{i=1}^{n}w_{i}X_{i}Y_{i}-{\bar{X}}_{w}{\bar{Y}}_{w}}{\sqrt{\sum_{i=1}^{n}w_{i}(X_{i}-{\bar{X}}_{w}{)}^{2}}\sqrt{\sum_{i=1}^{n}w_{i}(Y_{i}-{\bar{Y}}_{w}{)}^{2}}},$$

which may be expressed as ${\hat{\rho }}_{w}=g(V)$ where

(10) $$V=\left({\bar{X}}_{w},{\bar{Y}}_{w},\sum_{i=1}^{n}w_{i}X_{i}^{2},\sum_{i=1}^{n}w_{i}Y_{i}^{2},\sum_{i=1}^{n}w_{i}X_{i}Y_{i}\right)$$

and

(11) $$g(z_{1},z_{2},z_{3},z_{4},z_{5})=\frac{z_{5}-z_{1}z_{2}}{\sqrt{z_{3}-z_{1}^{2}}\sqrt{z_{4}-z_{2}^{2}}}.$$

By Theorem 3.1 of [7] the vector V is $\mathrm{AN}(E\{V\},\sum_{i=1}^{n}w_{i}^{2}\Sigma )$, where the 5×5 matrix Σ is the covariance matrix for $\left(X,Y,X^{2},Y^{2},\mathrm{XY}\right)$. It follows from standard theory pertaining to the transformations of asymptotic normally distributed variables, where

(12) $$d=\left(\frac{\partial g}{\partial z_{1}}{|}_{z=E(V)},\ldots ,\frac{\partial g}{\partial z_{5}}{|}_{z=E(V)}\right)$$

that (8) holds.

**Comment.** Similar to the unweighted case, if $F_{\mathrm{XY}}$ is bivariate normal (8) simplifies to ${\hat{\rho }}_{w}∼\mathrm{AN}(\rho ,\sum_{i=1}^{n}w_{i}^{2}(1-\rho^{2}{)}^{2})$.

Corollary 1 The asymptotic relative efficiency (A.R.E) of ${\hat{\rho }}_{w}$ to $\hat{\rho }$, for weights constrained throughout this note as $\sum_{i=1}^{n}w_{i}=1$ and $0<w_{i}<1,i=1,2,\ldots ,n$ is

(13) $$A.R.E=\frac{1/n}{\sum_{i=1}^{n}w_{i}^{2}}.$$

The A.R.E. result follows from  (8) and  (3), respectively.

As a simple illustration of the *A*.*R*.*E* suppose we had a sample of size *n* = 10, where the first five observations had $w_{i}=1/15$, i=1,2,…,5 and latter five observations had $w_{i}=2/15$, i=6,7,…,10. Then the A.R.E. of ${\hat{\rho }}_{w}$ to $\hat{\rho }$ is 90%. If we had a sample of size *n* = 10, where the first five observations had $w_{i}=1/20$, i=1,2,…,5 and latter five observations had $w_{i}=3/20$, i=6,7,…,10. Then the A.R.E. of ${\hat{\rho }}_{w}$ to $\hat{\rho }$ is 80%. In general, the use of the weighted correlation comes down to the weighting motivations, e.g. robustness considerations or other logical reasons, versus efficiency as compared to the unweighted Pearson correlation estimator.

### 2.2. Random weights

For the random weights case, we will utilize the work of Roozegar and Soltani [18] as a starting point. Let $S_{n}=\sum_{i=1}^{n}W_{i}X_{i}$ denote a randomly weighted average, where $X_{1},X_{2},\ldots X_{n}$ are a sequence of i.i.d. random variables, E(X)=0 and $E(X^{2})=\sigma^{2}<\infty$. In their work, Roozegar and Soltani [18] considered the more general unequal variance case per observation, which goes beyond our needs and hence we can reduce some of their notation to fit our goals. The work of Roozegar and Soltani [18] is focused on the case where the random weights $W_{i}$ are found by generating the uniform order statistic $U=(U_{\left(1\right)},U_{\left(2\right)},\ldots ,U_{\left(n-1\right)})$, where $U_{\left(1\right)}<U_{\left(2\right)}<\cdots <U_{\left(n-1\right)}$. Then the random weights are defined as $W_{i}=U_{\left(i\right)}-U_{\left(i-1\right)}$, i=1,2,3,…,n, where $U_{\left(n\right)}=1$ and $U_{\left(0\right)}=0$. Under the constant finite variance assumption above we start with the following:

**Lemma 2.5** [ [18] **(simplified for a constant variance)**]. Let $T_{n}=\sqrt{n+1}S_{n}$, where $S_{n}$ is defined above and $E(X^{2})=\sigma^{2}$ then

(14) $$T_{n}∼\mathrm{AN}(0,2\sigma^{2}),\;\text{as}\;n\to \infty .$$

See Ref. [18] for detailed proof based on the characteristic function of $T_{n}$ converging to the characteristic function of a normal distribution.

Denote the randomly weighted sample Pearson correlation estimator for *ρ* with random weights defined above as $W_{i}=U_{\left(i\right)}-U_{\left(i-1\right)}$, i=1,2,3,…,n, where $U_{\left(n\right)}=1$ and $U_{\left(0\right)}=0$ and $U_{\left(i\right)}$ is the *i*th uniform order statistic from a sample of size *n*−1 as follows:

(15) $${\hat{\rho }}_{w}=\frac{\sum_{i=1}^{n}W_{i}(X_{i}-{\bar{X}}_{W})(Y_{i}-{\bar{Y}}_{W})}{\sqrt{\sum_{i=1}^{n}W_{i}(X_{i}-{\bar{X}}_{W}{)}^{2}}\sqrt{\sum_{i=1}^{n}W_{i}(Y_{i}-{\bar{Y}}_{W}{)}^{2}}},$$

where ${\bar{X}}_{W}=\sum_{i=1}^{n}W_{i}X_{i}$, $\bar{Y}=\sum_{i=1}^{n}W_{i}Y_{i}$, $\sum_{i=1}^{n}W_{i}=1$ and $0<W_{i}<1,i=1,2,\ldots ,n$.

Theorem 2 For large *n* and ${\hat{\rho }}_{W}$ defined at (15), we have the following:

(16) $${\hat{\rho }}_{W}∼\mathrm{AN}(\rho ,\frac{2}{n}d\Sigma d^{\prime }),$$

where the elements of d are given at (4).

Proof. First, note that $\sqrt{\frac{n}{n+1}}\to 1$ as n→∞. The rest of the proof follows identically to Theorem 1 for fixed weights where the vector V is $\mathrm{AN}(E\{V\},\frac{2}{n}\Sigma )$ by Lemma 2.5 of [18]. The rests of the steps of the proof are identical to the fixed weights scenario where again the elements of the vector d are given at (4).

**Comment.** It follows from Theorem 2 that the A.R.E. of ${\hat{\rho }}_{W}$, with random weights defined as $W_{i}=U_{\left(i\right)}-U_{\left(i-1\right)}$, i=1,2,3,…,n, where $U_{\left(n\right)}=1$ and $U_{\left(0\right)}=0$ and $U_{\left(i\right)}$ is the *i*th uniform order statistic from a sample of size *n*−1, to that of $\hat{\rho }$ with fixed weights 1/*n* is 50%.

A more general statement about the large sample distribution for ${\hat{\rho }}_{W}$ defined at (15) may be made if similar to the fixed weight scenario we assume $\sum_{i=1}^{n}E(w_{i})=1$ and $0<E(w_{i})<1,i=1,2,\ldots ,n$. We then have the following:

Corollary 2.1 For large *n* and ${\hat{\rho }}_{W}$ defined at (15), we have the following:

(17) $${\hat{\rho }}_{W}∼\mathrm{AN}(\rho ,\sum_{i=1}^{n}E(W_{i}^{2})d\Sigma d^{\prime }),$$

where the elements of d are given at (4).

Proof. We basically follow the same basic steps and assumptions of [18] replacing dividing a U(0,1) distribution with a general absolutely continuous distribution having support (0,1) such that as stated above $\sum_{i=1}^{n}E(w_{i})=1$ and $0<E(w_{i})<1,i=1,2,\ldots ,n$.

### Variance estimators for ${\hat{\rho }}_{w}$ and ${\hat{\rho }}_{W}$

2.3.

In order to estimate the $\text{Var}({\hat{\rho }}_{w})$ one needs an estimator for the variance-covariance matrix Σ at (8), where as stated previously Σ is the 5×5 variance-covariance matrix for the vector $\left(X_{1},Y_{1},X_{1}^{2},Y_{1}^{2},X_{1}Y_{1}\right)$. Towards this end define the weighted moment estimators for E(X), E(Y), $E(X^{2})$, $E(Y^{2})$ and E(XY) in standard fashion as ${\bar{x}}_{w}=\sum_{i=1}^{n}w_{i}x_{i}$, ${\bar{y}}_{w}=\sum_{i=1}^{n}w_{i}y_{i}$, ${\bar{x^{2}}}_{w}=\sum_{i=1}^{n}w_{i}x_{i}^{2}$, ${\bar{y^{2}}}_{w}=\sum_{i=1}^{n}w_{i}y_{i}^{2}$ and ${\bar{\mathrm{xy}}}_{w}=\sum_{i=1}^{n}w_{i}x_{i}y_{i}$, respectively.

Similarly, define the moment estimators for Var(X), Var(Y), $\text{Var}(X^{2})$, $\text{Var}(Y^{2})$ and Var(XY) in standard fashion as $s_{x_{w}}^{2}=n\sum_{i=1}^{n}w_{i}(x_{i}-\bar{x_{w}}{)}^{2}/(n-1)$, $s_{y_{w}}^{2}=n\sum_{i=1}^{n}w_{i}(y_{i}-{\bar{y}}_{w}{)}^{2}/(n-1)$, $s_{x_{w}^{2}}^{2}=n\sum_{i=1}^{n}w_{i}(x_{i}^{2}-{\bar{x^{2}}}_{w}{)}^{2}/(n-1)$, $s_{y_{w}^{2}}^{2}=n\sum_{i=1}^{n}w_{i}(y_{i}^{2}-{\bar{y^{2}}}_{w}{)}^{2}/(n-1)$ and $s_{xy_{w}}^{2}=n\sum_{i=1}^{n}w_{i}(x_{i}y_{i}-{\bar{\mathrm{xy}}}_{w}{)}^{2}/(n-1)$, respectively. The n/(n−1) correction factor is so that the weighted moment estimators align with the unweighted case when all $w_{i}=1/n$.

Now for compactness of notation let $p_{i}=(x_{i}-{\bar{x}}_{w})/s_{x_{w}}$, $q_{i}=(y_{i}-{\bar{y}}_{w})/s_{y_{w}}$, $r_{i}=(x_{i}^{2}-{\bar{x^{2}}}_{w})/s_{x_{w}^{2}}$, $s_{i}=(y_{i}^{2}-{\bar{y^{2}}}_{w})/s_{y_{w}^{2}}$ and $t_{i}=(x_{i}y_{i}-{\bar{\mathrm{xy}}}_{w})/s_{xy_{w}}$. Also denote the diagonal n×n matrix

(18) $$W=\left(\begin{array}{ccccc} w_{1} & 0 & \cdots & \cdots & 0\\ 0 & w_{2} & \cdots & \cdots & 0\\ \vdots & \vdots & \ddots & \vdots & \vdots \\ 0 & 0 & \cdots & \cdots & w_{n} \end{array}\right).$$

Then the moment-based estimator for the variance-covariance matrix for $\left(X_{1},Y_{1},X_{1}^{2},Y_{1}^{2},X_{1}Y_{1}\right)$ is given as

(19) $$\begin{array}{rl} \hat{\Sigma } & =(\begin{array}{ccc} s_{x_{w}}^{2} & ns_{x}s_{y_{w}}p^{⊤}Wq/(n-1) & ns_{x_{w}}s_{x_{w}^{2}}p^{⊤}Wr/(n-1)\\ & s_{y_{w}}^{2} & ns_{y_{w}}s_{x_{w}^{2}}q^{⊤}Wr/(n-1)\\ & & s_{x_{w}^{2}}^{2} \end{array}\\ & \;\begin{array}{cc} ns_{x_{w}}s_{y_{w}^{2}}p^{⊤}Ws/(n-1) & ns_{x_{w}}s_{xy_{w}}p^{⊤}Wt/(n-1)\\ ns_{y_{w}}s_{y_{w}^{2}}q^{⊤}Ws/(n-1) & ns_{x_{w}}s_{xy_{w}}q^{⊤}Wt/(n-1)\\ ns_{x_{w}^{2}}s_{y_{w}^{2}}r^{⊤}Ws/(n-1) & ns_{x_{w}^{2}}s_{xy_{w}}r^{⊤}Wt/(n-1)\\ s_{y_{w}^{2}}^{2} & ns_{y_{w}^{2}}s_{xy_{w}}s^{⊤}Wt/(n-1)\\ & s_{xy_{w}}^{2} \end{array}), \end{array}$$

where the elements 5×1 vectors p, q, r, s and t are provided in the paragraph above for i=1,2,…,n. It follows that

(20) $$\hat{\text{Var}({\hat{\rho }}_{w})}=\sum_{i=1}^{n}w_{i}^{2}d\hat{\Sigma }d^{\prime },$$

where $\hat{\Sigma }$ is given at (19) and the elements of d are defined at (4).

**Comment.** For the estimator ${\hat{\rho }}_{w}$ based on random weights, we can use the same variance estimator at (20) in that the observed $W_{i}$'s are in essence moment estimators for $E(W_{i})$'s.

## Permutation testing about $H_{0}:\rho =0$

3.

Diciccio and Romano [5] provide the theoretical justification in the unweighted case for when the straightforward permutation test about $H_{0}:\rho =0$ is exact, i.e. when it is equivalent to testing $H_{0}:F_{\mathrm{XY}}=F_{X}F_{Y}$ under exchangeability assumptions holding. They then note that when the permutation test for $H_{0}:\rho =0$ is not equivalent to testing $H_{0}:F_{\mathrm{XY}}=F_{X}F_{Y}$ the Type I error may be either dramatically over or underinflated conditional upon the dependence structure of $F_{\mathrm{XY}}$. The correction to this issue is the proper standardization of the test statistic $\sqrt{n}\hat{\rho }$ under permutations such that a test is constructed that asymptotically may be shown to control the Type I error rate at the desired level.

We follow the same prescription as Diciccio and Romano [5] for the weighted sample Pearson correlation as per the unweighted case. First, define $G_{n}$ to be the set of all permutations *π* of {1,…,n} for testing independence between two random variables *X* and *Y*. Then the permutation distribution of any given weighted test statistic $T_{n,w}(X^{n},Y^{n})$ is defined as

(21) $${\hat{R}}_{n}^{T_{n,w}}(t)=\frac{1}{n!}\sum_{\pi \in G_{n}}I\{T_{n,w}(X^{n},Y_{\pi }^{n})\leq t\}$$

where $Y_{\pi }^{n}$ represents a given permutation $\left\{Y_{\pi (1)},\ldots ,Y_{\pi (n)}\right\}$. In this setting, the permutation $G_{n}$ is all possible pairwise combinations between $\left(w^{n},X^{n}\right)$ and $Y^{n}$. The key is to note in the weighted setting is that the form $T_{n,w}(X^{n},Y^{n})$ assumes fixed weights such that for each permutation we define the test statistic to have the form

(22) $$T_{n,w}(X^{n},Y_{\pi }^{n})=\sum_{i=1}^{n}w_{i}g(X_{i}^{n},Y_{i,\pi }^{n}),$$

i.e. the weights, $w_{i}$, i=1,2,…,n, are held fixed per each permutation. For testing $H_{1}:\rho >0$, the null hypothesis will be rejected if the test statistics is larger than the 1−α quantile of the permutation distribution. The test is exact under the exchangeability assumption. In the weighted setting, it requires the distribution of $\left((w^{n},X^{n}),Y^{n}\right)$ is invariant under the group of transformations $G_{n}$.

The test using the statistics $T_{n,w}(X^{n},Y^{n})={\hat{\rho }}_{w}$ is exact when testing $H_{0}:\rho =0$ is equivalent to testing

$$H_{0}:F_{\mathrm{XY}}=F_{X}F_{Y},$$

where $F_{X}$ and $F_{Y}$ are marginal distributions, i.e. exchangeability holds, see Theorem 6 Lehmann (1986). This equivalence holds for underlying distributions such as the bivariate normal distribution and other elliptically contoured distributions.

The weighted Pearson correlation permutation test follows the same identical algorithm as per the unweighted case as derived by Diciccio and Romano [5]. When testing $H_{0}:\rho =0$ is not equivalent to testing $H_{0}:F_{\mathrm{XY}}=F_{X}F_{Y}$ start by first assuming $E(X^{2})<\infty ,E(Y^{2}<\infty )$ and $E(X^{2}Y^{2})<\infty$. Then the permutation distribution of $S_{n,w}=\sqrt{\sum_{i=1}^{n}w_{i}^{2}}{\hat{\rho }}_{w}/\tau$ in the case of fixed weights satisfies

(23) $$\lim_{n\to \infty }\underset{t\in R}{\sup}|{\hat{R}}_{n}^{S_{n,w}}(t)-\Phi (t)|=0,$$

almost surely, where, Φ(t) is the standard normal c.d.f., $\sum_{i=1}^{n}w_{i}=1$ and $0<w_{i}<1,i=1,2,\ldots ,n$, ${\hat{R}}_{n}^{S_{n,w}}(t)$ is the permutation distribution for $S_{n},$ $\tau^{2}=\frac{\mu_{2,2}}{\mu_{0,2}\mu_{2,0}}$ where $\mu_{r,s}=E[(X-\mu_{X}{)}^{r}(Y-\mu_{Y}{)}^{s}]$. The results follow straightforward from Theorem 1 and given $H_{0}:\rho =0$ true. The same large sample result holds for random weights under the conditions of Theorem 2 and given $S_{w,n}=\sqrt{2/n}{\hat{\rho }}_{w}/\tau$.

**Comment.** Practically speaking there is no difference between the behavior of the permutation test for $H_{0}:\rho =0$ given fixed or random weight since for the fixed weight procedure $\sum_{i=1}^{n}w_{i}^{2}$ is a fixed constant/scalar for the observed ${\hat{\rho }}_{w}$ and its permuted value given the form (22). The behavior of the permutation test of $H_{0}:\rho =0$ based on ${\hat{\rho }}_{w}$ in terms of Type I error control will be examined extensively in the next section.

Note that we can use either the variance estimator in Equation (20) or the form derived by DiCiccio or Romano [5] for studentization. We denote the first test as Perm-1 and the second as Perm-2. The large sample variance is a function of *ρ*. Equation (20) is equivalent to plugging in ${\hat{\rho }}_{w}$, while the formula by DeCiccio and Romano can be obtained by substituting ${\hat{\rho }}_{w}$ with 0 (Perm-2).

More generally, we can define the weights in permutation samples as a function of the original weights and the permutation,

(24) $$w^{*}=f(w,\pi ),\sum_{i=1}^{n}w_{i}=1,$$

where *w* is the vector of original weights, and $w^{*}$ is the weights under permutation. One example of f(w,π) is to generate a new weight for each of the permuted observation based on the average of the original weights of corresponding *X* and *Y*. Namely, assume the sample after permutation is $\left(X^{n},Y_{\pi }^{n}\right)$, then the weight will be

$$f(w,\pi )=\frac{1}{2}(w+w_{\pi }).$$

We denote this test as Perm-ave.

Finally, since the variance estimation relies on large sample approximation, we postulate a test based on Fisher's *Z*-transformation of *ρ* may further improve the type I error control. The transformed statistic is defined as, $Z=-\frac{1}{2}\log\frac{1+{\hat{\rho }}_{w}}{1-{\hat{\rho }}_{w}}$ and its large sample variance can be approximated by $\left(1-\rho^{2})\mathrm{Var}(\rho \right)$. Similarly, two variance estimators can be obtained by substituting *ρ* with ${\hat{\rho }}_{w}$ (Perm-Z1) or 0 (Perm-Z2).

## Permutation testing about $H_{0}:\rho =\rho_{0}$

4.

More generally, we may be interested in testing $H_{0}:\rho =\rho_{0}$ versus $H_{1}:\rho >\rho_{0}$, for which a conventional permutation test cannot be directly applied. In this case, the asymptotic test based large sample approximation will be a natural option, though it may have an inflated type I error rate when *n* is small. Alternatively, a permutation test about non-zero correlations under the null can be achieved by using a de-correlated sample, as introduced by [13]. This approach ensures type I error control for testing the non-zero concordance correlation coefficient even when *n* is as small as 10.

We adopt this method in testing the non-zero null hypothesis. Specifically, following [13], the original observations are standardized by

$$U_{i}^{\prime }=\frac{X_{i}-\bar{X}}{S_{X}},V_{i}^{\prime }=\frac{Y_{i}-\bar{Y}}{S_{Y}},$$

such that the $U_{i}^{\prime }$ and $V_{i}^{\prime }$ will have zero means and unit variances. The new variables can be then de-correlated by

$$(U,V{)}^{T}=A(\rho_{0})(U^{\prime },V^{\prime }{)}^{T},$$

where A(⋅) is defined as

$$A(x)=\left(\begin{array}{cc} 1 & 0\\ \frac{-x}{\sqrt{1-x^{2}}} & \frac{1}{\sqrt{1-x^{2}}} \end{array}\right),$$

which satisfies

$$A(\rho )\left(\begin{array}{cc} 1 & \rho \\ \rho & 1 \end{array}\right)A(\rho {)}^{T}=I_{2}.$$

Thus we have ρ(U,V)→0 under $H_{0}$. Therefore, the original hypotheses are transformed to $H_{0}:\rho (U,V)=0$ versus $H_{1}:\rho (U,V)>0$. The testing procedure will follow that in Section 3, but applied to *U* and *V*.

## 5. Simulations

### Test for $H_{0}:\rho =0$ versus $H_{1}:H_{0}:\rho >0$ via the weighted sample Pearson correlation coefficient

5.1.

We examined the Type I error control using the same distribution scenarios from DiCiccio and Romano [5], which was also utilized in the later work by Hutson and Yu [13]. An additional bivariate uniform distribution was added to the simulations. For our simulation, we focused on one-sided alternative, $H_{0}:\rho =0$ versus $H_{1}:\rho >0$. The sample sizes studied are n=10,20,50,100,200. Each simulation utilized 10, 000 Monte Carlo replications. For the permutation tests, the number of permutations was 1000. The weights were generated as $W_{i}=U_{\left(i\right)}-U_{\left(i-1\right)}$ and $W_{n}=1-\sum_{i=1}^{n-1}W_{i}$, where $U_{\left(i\right)}$ is the order statistic of a random sample $U_{1},\ldots ,U_{n-1}$ from beta distribution B(α,β). Here, we examined B(1,1), B(0.5,0.5) and B(3,3). Under each scenario, a set of weights was generated for every 1000 replications. We compared the straight *t*-test, the test based on large sample approximation by Equation (20) (Asymptotic) and studentized permutation test (Stu Perm). The details of these six simulation scenarios are listed below,

1. Standard bivariate normal distribution with ρ=0.
2. Exponential. We define $(X,Y)=rS^{T}u$, where $S=\mathrm{diag}(\sqrt{2},1)$, *u* is uniformly distributed on the two-dimensional circle with a radius of 1 and *r* follows an exponential distribution with λ=1.
3. Circular. *X* and *Y* follow a uniform distribution on a two-dimensional circle with a radius of 1, such that $X^{2}+Y^{2}=1$.
4.
$t_{4.1}$. Let *W* and *Z* be random variables following iid *t*-distributions with 4.1 degrees of freedom, the *X* and *Y* are defined as *X* = *W* + *Z* and *Y* = *W*−*Z*, such that *X* and *Y* are dependent but with the covariance of 0.
5. Multivariate *t*-distribution with zero means, identity covariance and 5 degrees of freedom (MVT).
6. Uniform with *X* and *Y* following independent Uniform(0,1).

**Figure 1.**
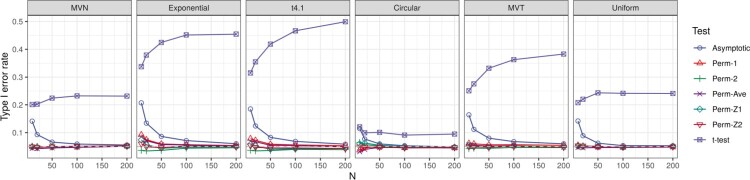
Type I errors for tests of $H_{0}:\rho =0$ versus $H_{1}:\rho >0$ with weights generated under Beta(1,1).

**Figure 2.**
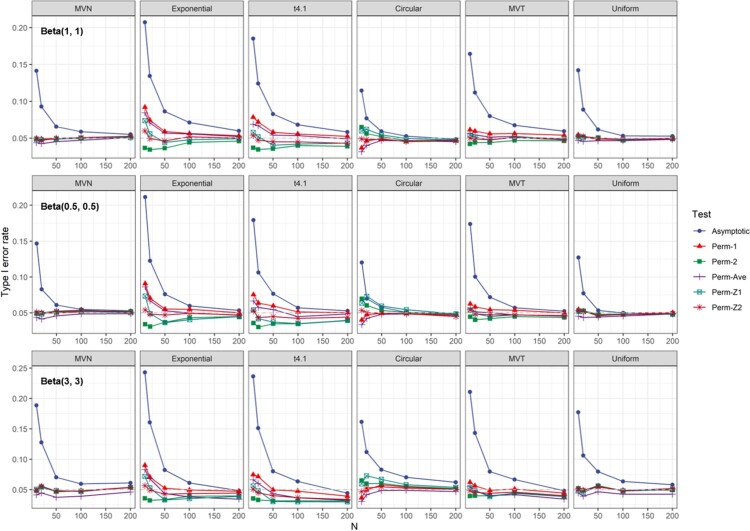
Type I errors for tests of $H_{0}:\rho =0$ versus $H_{1}:\rho >0$.

The type I error rates of the *t*-test are severely inflated in all cases (Figure 1), and hence are not included in the subsequent results. The results for testing $H_{0}:\rho =0$ are shown in Figure 2. Specifically, the asymptotic test tends to have an inflated type I error when *n* is small, but the error rates converge to 0.05 when *n* is as large as 50. The permutation tests all have similar and robust type I error controls. However, the Perm-2 and Perm-Z1 test tends to be conservative under Exponential and $t_{4.1}$ when *n* is small. On the other hand, Perm-1 and Perm-ave tend to have inflated rates under these two distributions. The Perm-Z2 test, which is based on Fisher transformation and variance estimator plugged in ρ=0, tends to have the most robust type I error control.

**Figure 3.**
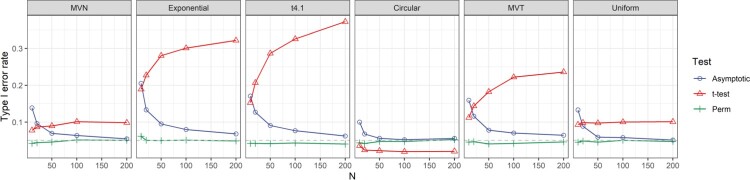
Type I errors for tests of $H_{0}:\rho =0$ versus $H_{1}:\rho >0$, when the weights are proportional to $1-h_{\mathrm{ii}}$.

We also examined a scenario where the weights are random and meanwhile functions of the data, which were defined based on the leverage when regressing *Y* on *X*. Specifically, we define $W_{i}$ as the $1-h_{\mathrm{ii}}$, where $h_{\mathrm{ii}}$ is the *i*th diagonal element of $H=X(X^{T}X{)}^{-1}X^{T}$. The weights were normalized so that $\sum_{i=1}^{n}W_{i}=1$. Figure 3 shows that even when the weights are functions of data, the studentized permutation test (Perm-Z2) still robustly controls the type I error under all scenarios investigated.

### Test for $H_{0}:\rho =\rho_{0}$ versus $H_{1}:H_{0}:\rho >\rho_{0}$ via the weighted sample Pearson correlation coefficient

5.2.

In this section, we examined whether the asymptotic test and and Perm-Z2 can also robustly control the type I errors when $\rho_{0}\neq 0$. The same settings as in Section 5.1 were used. We examined the weights generated based on B(1,1), because similar results are expected under different weight distributions. To generate the data with desired correlation, the *X* and *Y* were first standardized by the population standard deviations respectively. The standardized observations were then transformed through

$$B(\rho )=\left(\begin{array}{cc} 1 & 0\\ \rho & \sqrt{1-\rho^{2}} \end{array}\right),$$

where *ρ* is the target correlation.

**Figure 4.**
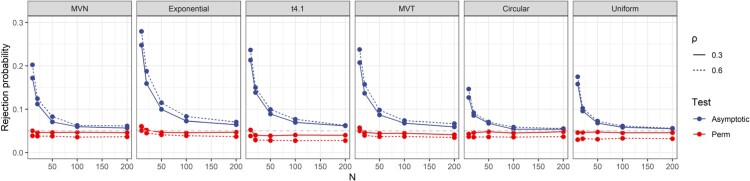
Type I errors for tests of $H_{0}:\rho =\rho_{0}$ versus $H_{1}:\rho >\rho_{0}$ when $\rho_{0}=0.3$ or 0.6.

Similar to what was presented in Section 5.1, the results in Figure 4 show that the asymptotic test tends to have an inflated type I error when *n* is small, but the error rates converge to 0.05 when *n* is large. The Perm-Z2 test still shows robust type I error control. The test tends to be slightly conservative under certain scenarios, e.g. $t_{4.1}$ and Uniform when $\rho_{0}=0.6$. These results suggest the asymptotic variance and its estimation is still valid when the underlying correlation is non-zero. The modified permutation tests can also maintain the type I error control under this scenario.

### 5.3. Power of test via the weighted sample Pearson correlation coefficient

**Figure 5.**
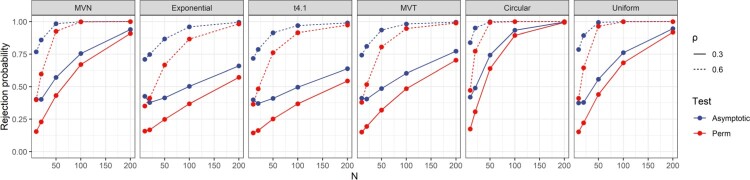
Power of tests of $H_{0}:\rho =0$ versus $H_{1}:\rho >0$ when the true ρ=0.3 or 0.6.

In this section, we examined the power of asymptotic test and Perm-Z2 for the tests of $H_{0}:\rho =0$ versus $H_{1}:\rho >0$. The same simulation settings as in Section 5.2 were used. Figure 5 shows the results when the true *ρ* is 0.3 and 0.6. The power of asymptotic test is generally higher than that of Perm-Z2. However, it should be noted that the type I error rates of asymptotic tests generally suffer severe inflations when n≤50. Therefore, the power of the two tests is not comparable when *n* is small. When n≥100, the difference in power between the two tests is small under the settings of MVN, Circular and Uniform. On the other hand, such difference can be large under Exponential, $t_{4.1}$ and MVT settings when ρ=0.3. Based on these results, a recommendation is to use a permutation test when the sample size is small, e.g. n≤50. When *n* is larger, the asymptotic test will provide higher power while still maintaining decent type I error control in general.

## 6. Example

To demonstrate the proposed method, we applied it to a data set of preterm birth, stillbirth and neonatal death rates from 28 countries [15]. In the original work, the authors studied the correlations of preterm birth rates with stillbirth and neonatal death rates at the country level. The correlations were weighted by the total live births in each country. The preterm births were categorized as <37, 32–36, 28–31, 24–27 weeks of gestation, while the stillbirths and neonatal deaths were categorized into ≥32 and ≥37 weeks. Only point estimates were provided for the weighted correlations in the original work. For demonstration purposes, we estimated all pairwise weighted correlations between preterm birth, stillbirth and neonatal death rates in each category. The *p*-values of one-sided tests by *t*-test, the test based on Jackknife variance estimation, asymptotic tests and Perm-Z2 were compared (Table 1). Directions of the tests were determined by the sign of ${\hat{\rho }}_{w}$.

**Table 1.** The estimates of weighted correlations of preterm birth, stillbirth and neonatal death rates at country levels (*n* = 28).

Rate 1	Rate 2	${\hat{\rho }}_{w}$	*t*-test	Jackknife	Asymptotic	Perm Z2
Preterm ( <37)	Stillbirth ( ≥32)	−0.6973	<**0**.**0001**	0.0680	0.0562	**0**.**0220**
Preterm ( <37)	Stillbirth ( ≥37)	−0.7134	<**0**.**0001**	0.0652	**0**.**0410**	**0**.**0130**
Preterm ( <37)	Neonatal ( ≥32)	−0.1675	0.2171	0.3329	0.2879	0.3460
Preterm ( <37)	Neonatal ( ≥37)	−0.2716	0.0996	0.2313	0.1745	0.2380
Preterm (32−36)	Stillbirth ( ≥32)	−0.7259	<**0**.**0001**	**0**.**0472**	**0**.**0355**	**0**.**0160**
Preterm (32−36)	Stillbirth ( ≥37)	−0.7360	<**0**.**0001**	**0**.**0470**	**0**.**0243**	**0**.**0080**
Preterm (32−36)	Neonatal ( ≥32)	−0.1896	0.1874	0.3077	0.2802	0.3520
Preterm (32−36)	Neonatal ( ≥37)	−0.2969	0.0794	0.2084	0.1616	0.2550
Preterm (28−31)	Stillbirth ( ≥32)	−0.4207	**0**.**0129**	0.2281	0.2211	0.2100
Preterm (28−31)	Stillbirth ( ≥37)	−0.4576	**0**.**0072**	0.2088	0.2089	0.1740
Preterm (28−31)	Neonatal ( ≥32)	−0.1151	0.2962	0.3718	0.4175	0.3750
Preterm (28−31)	Neonatal ( ≥37)	−0.1660	0.2190	0.2830	0.3806	0.3360
Preterm (24−27)	Stillbirth ( ≥32)	−0.5116	**0**.**0027**	0.1795	0.1645	0.1290
Preterm (24−27)	Stillbirth ( ≥37)	−0.5775	**0**.**0006**	0.1441	0.1497	0.0800
Preterm (24−27)	Neonatal ( ≥32)	−0.0566	0.3965	0.4010	0.4435	0.4330
Preterm (24−27)	Neonatal ( ≥37)	−0.1470	0.2465	0.3357	0.3601	0.3090
Stillbirth ( ≥32)	Neonatal ( ≥32)	0.4833	**0**.**0084**	**0**.**0156**	0.0663	0.1750
Stillbirth ( ≥32)	Neonatal ( ≥37)	0.6132	**0**.**0007**	**0**.**0161**	**0**.**0160**	0.1140
Stillbirth ( ≥37)	Neonatal ( ≥32)	0.4075	**0**.**0240**	**0**.**0430**	**0**.**0331**	0.2290
Stillbirth ( ≥37)	Neonatal ( ≥37)	0.5749	**0**.**0016**	**0**.**0408**	**0**.**0001**	0.1310

Note: The *p*-values are based on one-sided tests. The statistically significant *p*-values are shown in bold ( α=0.05).

The results (Table 1) show that all three tests will conclude the preterm birth rate ( <37 weeks) is negatively correlated with the stillbirth rate ≥37 weeks. Further, the preterm rates between 32 and 36 weeks are negatively correlated with both early and late stillbirth rates. However, there are discrepancies among the three methods. For example, the late stillbirth rates ( ≥37 weeks) are considered significantly associated with the neonatal rates by both the *t*-test and asymptotic test, but not by the permutation test. This is consistent with the observation in simulations that the *t*-test tends to have an inflated type I error in general while the asymptotic test tends to have an inflated type I error when the sample size is below 50. It is also notable that the correlation between preterm <37 weeks and stillbirth ≥32 weeks was considered significant by both the *t*-test and permutation test but not by the asymptotic test. Therefore, these three tests could lead to distinct conclusions in applications. Based on our numeric results, the result of permutation test is clearly more reliable.

## 7. Concluding remarks

In this note, we introduced an asymptotic test and a set of studentized permutation tests for testing the hypothesis $H_{0}:\rho =0$ vs. $H_{1}:\rho >0$. We showed that the *t*-test routine that is implemented in widely used software like SAS could lead to severe type I error inflation even when the data is bivariate normal. The proposed asymptotic test tends to have an inflated type I error when the sample size is smaller than *n* = 50, which is similar to the unweighted cases. We found that the studentized permutation test based on the Fisher's *Z* statistic is the most robust, even when the sample is small and the data is non-normal. Note that the choice of statistic affects the result of permutation test because the statistic is studentized by its large sample variance. We also showcase that the *t*-test and proposed tests can lead to a distinct conclusion in real-world applications. The *t*-test will yield a large number of false positives regardless of the underlying distribution. Therefore, we conclude that the routine usage of *t*-test in conjunction with the weighted sample Pearson correlation should be removed from statistical practice and eliminated from statistical software packages. On the other hand, the studentized permutation tests have strong type I error control even for very small sample sizes while the asymptotic approximation appears valid for *n*>50.
